# Using SAS Macros for Multiple Mediation Analysis in R

**DOI:** 10.5334/jors.277

**Published:** 2020-10-07

**Authors:** Paige Fisher, Wentao Cao, Qingzhao Yu

**Affiliations:** Louisiana State University Health Sciences Center, New Orleans, US

**Keywords:** Mediation Analysis, Confounding Analysis, SAS Macros, R package mma

## Abstract

Mediation analysis refers to the process of making inferences on effects of third variables that intervene in the relationship between an exposure and response variable. The relationships among variables can be modelled by generalized linear models (GLM). However, GLM are not sufficient to describe relationships among variables when there are nonlinear relationships and potential interaction effects. A general mediation analysis method was developed using not only GLMs, but also multiple additive regression trees and smoothing splines by Yu and Li (2017). The method is implemented in the R package, *mma*. In this paper, we developed SAS macros so that functions in the mma package can be called and the mediation analysis performed in the SAS environment.

## (1) Overview

### Introduction

A third variable can intervene the relationship between an exposure and response variable through the pathway where the exposure variable affects the third variable, which in turn, affects the response variable. The third variable is called a mediator or an intervening variable, denoted as M. We further denote the exposure variable as X, and the response variable as Y. Mediation effects include indirect and direct effect. The indirect effect measures how much of the association between X and Y can be explained through the X ➔ M ➔ Y pathway. Yu and Li [1] compiled a R package mma, which uses generalized linear regressions and nonlinear methods to model relationships among variables, based on which inferences on mediation effects can be drawn. For the concepts and estimation algorithm of mediation effects, please refer to [1], [2], and [3].

We developed SAS macros for SAS users so that the multiple mediation analysis can be performed utilizing the R package, “mma” [1] in the SAS environment. With the mma package, mediation analysis is done through three functions: 1. data.org is used to identify potential mediators and transform the data set into an analytic form; 2. the function *med* generates estimates of mediation effects based on the whole data set; and 3. the function *boot.med* calculates variances and confidence intervals for estimates of mediation effects using bootstrap methods. In addition, the “mma” package provides visual aids and summary functions to help users understand mediation effects. The *plot* function is used to illustrate the directions of mediation effects. In this paper, we present SAS macros that call each function from SAS to perform the mediation analysis and report results. SAS users can implement the mediation analysis within the SAS environment. No knowledge of R is needed for the analysis. Furthermore, analysis results from R are imported to SAS automatically. SAS users can perform further analysis using tools provided by SAS (e.g. graphical tools) for their specific aims of research.

### Implementation and architecture

#### Running R in SAS

In order to run R packages in SAS, SAS version 9.22 or above is required. To communicate with R, a RLANG option must be set when SAS is started. This is set through modifying the sasv9.cfg file. To edit this file, users must run the operating system (i.e. Windows) as an administrator. The file is usually located in the path C:\Program Files\SASHome\x86\SASFoundation\9.4. Once the file is located, three lines are to be edited as follows. Change the file path when necessary.


-RLANG
-config “C:\Program Files\SASHome\x86\SASFoundation\9.4
    \nls\en\sasv9.cfg”
-SET R_HOME “C:\Program Files\R\R-3.4.3”


Usually users do not have to change the second line. The first line and third line needs to be added. The location and the version of R in the third line need to be modified according to the users’ R setting.

Next, right click on the SAS desktop icon and select “Properties” and add –RLANG to the end of the target command line. And then, right click on the SAS icon and choose “run as administrator”. Lastly, run the following command in SAS:

proc options option = RLANG value; run;.


In the SAS log a message appears that states:

SAS (r) Proprietary Software Release 9.4 TS1M1
Option Value Information For SAS Option RLANG
Value: RLANG
Scope: SAS Session
How option value set: SAS Session Startup Command Line


When RLANG appears, congratulations, the macro is able to run in SAS. For more information about calling R within SAS, readers are referred to [4].

#### Proc_R_dataorg Macro

The function data.org is used to identify potential mediators and to transform data sets into the analytic form. To be identified as a mediator, the variable should be significantly correlated with the predictor and the variable must be significantly related to the outcome, given that all other related variables are adjusted for in the model. If a variable meets both conditions, it will be included as a mediator in the dataset. If a variable is only significantly related to the outcome, then the variable is included in the dataset as a covariate but not a mediator. The argument JOINTM identifies groups of mediators, where the joint effect of all variables in each group is of interest. The individual and joint mediation effect of a group of mediators are reported separately. All variables identified in JOINTM are forced to enter the model as mediators without being tested for significant relationships.

### Arguments

Proc_R_dataorg is a macro that calls the data.org function in the mma package from SAS. It is included in the supplementary file Proc_R_dataorg.sas. The arguments that are needed in the macro are summarized in Table 1. More information on how to define the arguments MEDIATOR, CONTMED, BINMED, BINREF, CATMED, CATREF, PREDREF, JOINTM, REFY, ALPHA, ALPHA2, X, PRED, Y, TIME, STATUS can be found in [3].

### Implementation

The user first runs the %INCLUDE statement with the location of the macro file Proc_R_dataorg.sas. All arguments in Table 1 can be defined and saved in the temp_setup_mma_macro.sas file, as in the example. The user must save the macro programs Proc_R_dataorg.sas and R_submit_dataorg.sas in the appropriate location(s) defined in the argument PATH_R. Before the data.org function in R is executed, the commands in the R_submit_dataorg.sas macro generates a text file from the original SAS dataset and then have it read into R.

After R is initiated, the mma library is loaded and the text file, which was created and stored in the location PATHD, is read into R, and then the data.org function is executed. Results generated from data.org is stored in the data.bin.RData file and as a text file in data_bin.txt, in the location defined by the argument PATH_R.

### Example

#### The dataset

In the example, the data set “*weight_behavior*” is used. The set contains survey data collected from selected children, teachers and parents in Grenada in 2014 to explore how kids behavior variables explain the gender differences in overweight [1]. *Weight_behavior* includes 691 observations and 15 variables.

In this example, the data set is saved as a SAS dataset and stored in C:\myfolder\data directory.

#### Implementation

The code below defines all macro arguments used to run the Proc_R_dataorg macro for this example.

Firstly, users define the arguments with the following template (setup_mma_macroe_wb.sas). Note that all templates with comments are provided as supplementary material with this paper.

The following SAS macro arguments are defined to identify mediators and covariates that explain the gender difference in being overweight using the *weight_behavior* data set. The outcome variable, y, is the binary variable *overweight* and the predictor, x, is *sex* where males are the reference group. The continuous variables *gotosch, snack, tvhours, cellhours, sports*; and binary variables *car, cmpthours*; and multi-categorical variable *numpeople*, are all potential mediators.


libname lib “C:\myfolder\data” ;
%let path= C:\myfolder ;
%let pathd=C:\myfolder\data ;
%let data=weight_behavior ;
%let pre=lib.;
%let path_r=C:/myfolder ;
%let x=%str(data[,c(2,4:14)]) ;
%let pred=%str(data[,3]) ;
%let y=%str(data[,15]) ;
%let contmed=c(7:9,11:12) ;
%let binmed=c(6,10) ;
%let binref=c(1,1) ;
%let catmed=5 ;
%let catref=1 ;
%let predref=M ;
%let alpha=0.4 ;
%let alpha2=0.4 ;


Once the SAS macro arguments are defined, execute the macro by the %INCLUDE statement in SAS


%include “&path\Proc_R_dataorg.sas”;


Lastly, run the Proc_R_dataorg macro.


%Proc_R_dataorg(&pre, &path_r, &data, &mediator, 
&contmed, &binmed, &binref, &catmed, &catref, 
&predref, &refy, &alpha, &alpha2, &x, &pred, &y);


#### Results

The function data.org creates a data list with x, dirx, y, and fullmodel. The data set, x, includes all identified mediators and covariates in explaining the outcome which is defined in y; dirx defines the predictor.

**Figure 1:**
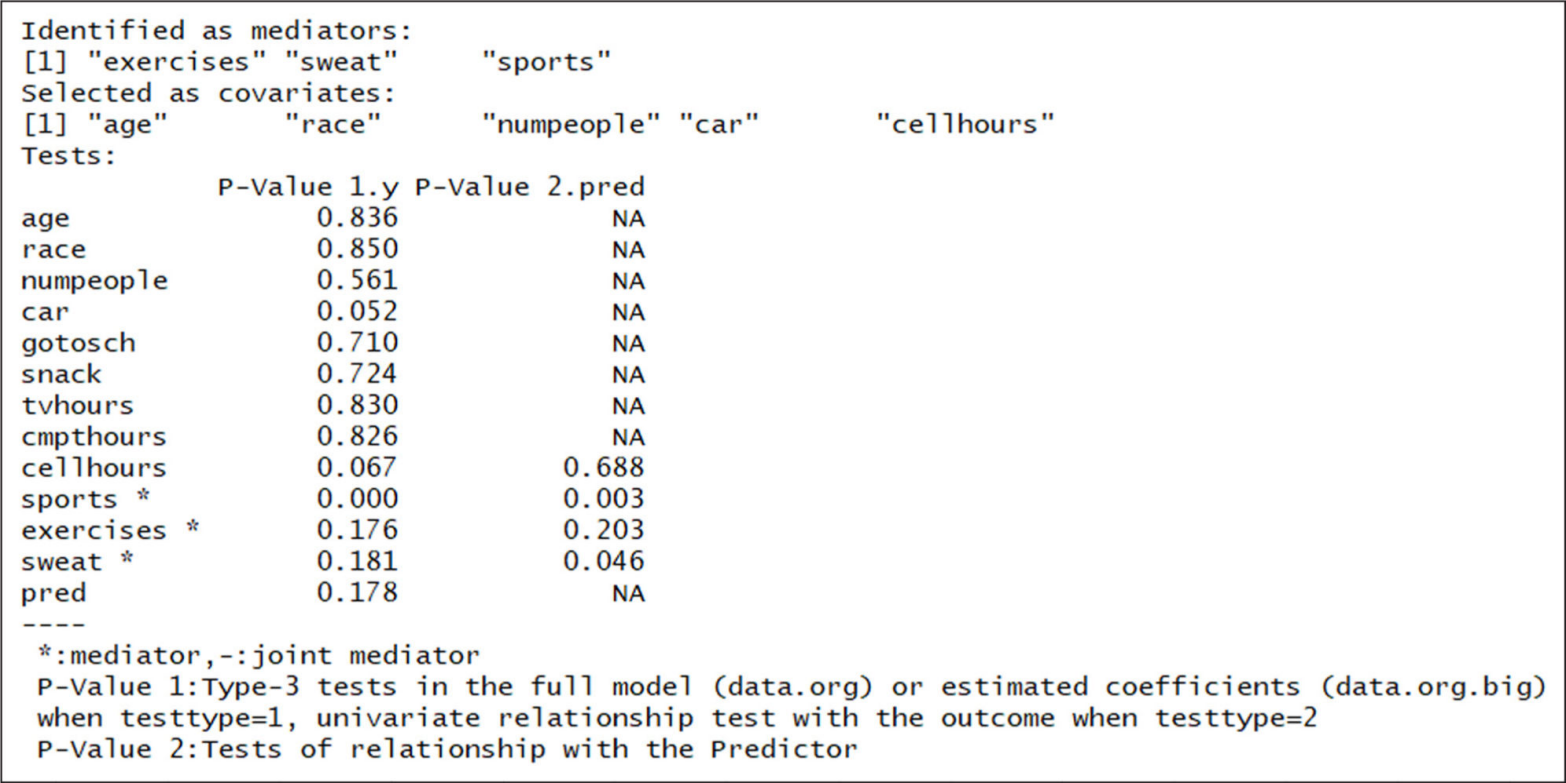
Summary of identified mediators.

Figure 1 shows the results of calling the macro. In this example, the variables “exercises”, “sweat”, and “sports” were identified as mediators and “age”, “race”, “numpeople”, “car” and “cellhours” were selected as covariates. P-value 1 is the test result for the relationship between the corresponding variable with the outcome. P-value 2 is for the relationship between the exposure variable and the potential mediator.

#### Proc_R_med Macro

The function med is used to estimate the direct effect of the exposure variable and indirect effects of each mediator through mediation analysis with the identified mediators and covariates from Proc_R_dataorg Macro.

### Arguments

The Proc_R_med macro is included in the file Proc_R_med.sas. The arguments used for the macro but not in Table 1 are summarized in Table 2.

More information on how to define the arguments REFY, MARGIN, D, DISTN, n, NU, NONLINEAR, DF1, TYPE can be found in [3].

### Implementation

The arguments in Table 2 are defined in the temp_setup_mma_macro.sas file. The user must run the %INCLUDE statement which indicates the location of the macro program files. Two macro programs, Proc_R_med.

sas and R_submit_med.sas, need to be stored in the appropriate location(s) defined in the argument PATH_R. The Proc_R_med.sas program calls the macro arguments that were defined in the temp_setup_mma_ macro.sas file and executes the R_submit_med.sas program that calls the R med function from SAS. The macro first calls in data that was created by the Proc_R_dataorg macro, data.bin.RData, and then conducts mediation analysis with the identified mediators and covariates.

The result is a med object and saved as a R dataset, temp.med.Rdata. The data contains a matrix *denm* with the estimated direct effect, a matrix *ie* with the estimated indirect effect from each mediator, a vector *te* with the estimated total effect on the exposure variable, and the final full model results. The macro also prints out the model results in the SAS results window.

### Example

#### The dataset

Using the same example as above, the data.bin.RData that was exported from the Proc_R_dataorg macro will be used.

#### Implementation

The code below defines all of the macro arguments used to run the Proc_R_med macro for this example.

To calculate the indirect effects, the following codes request resampling 2 times for estimation.


%let rdata=data.bin ;
%let n=2 ;
%let nonlinear=FALSE ;


Now, execute the %INCLUDE statement to run the SAS program which contains the macro.


%include “&path\Proc_R_med.sas” ;


Lastly, run the Proc_R_med macro.


%Proc_R_med(&pre,&path_r,&rdata,&margin,&D,&dist 
n,&refy,&n,&nu,&nonlinear,&df1,&type)


#### Results

**Figure 2:**
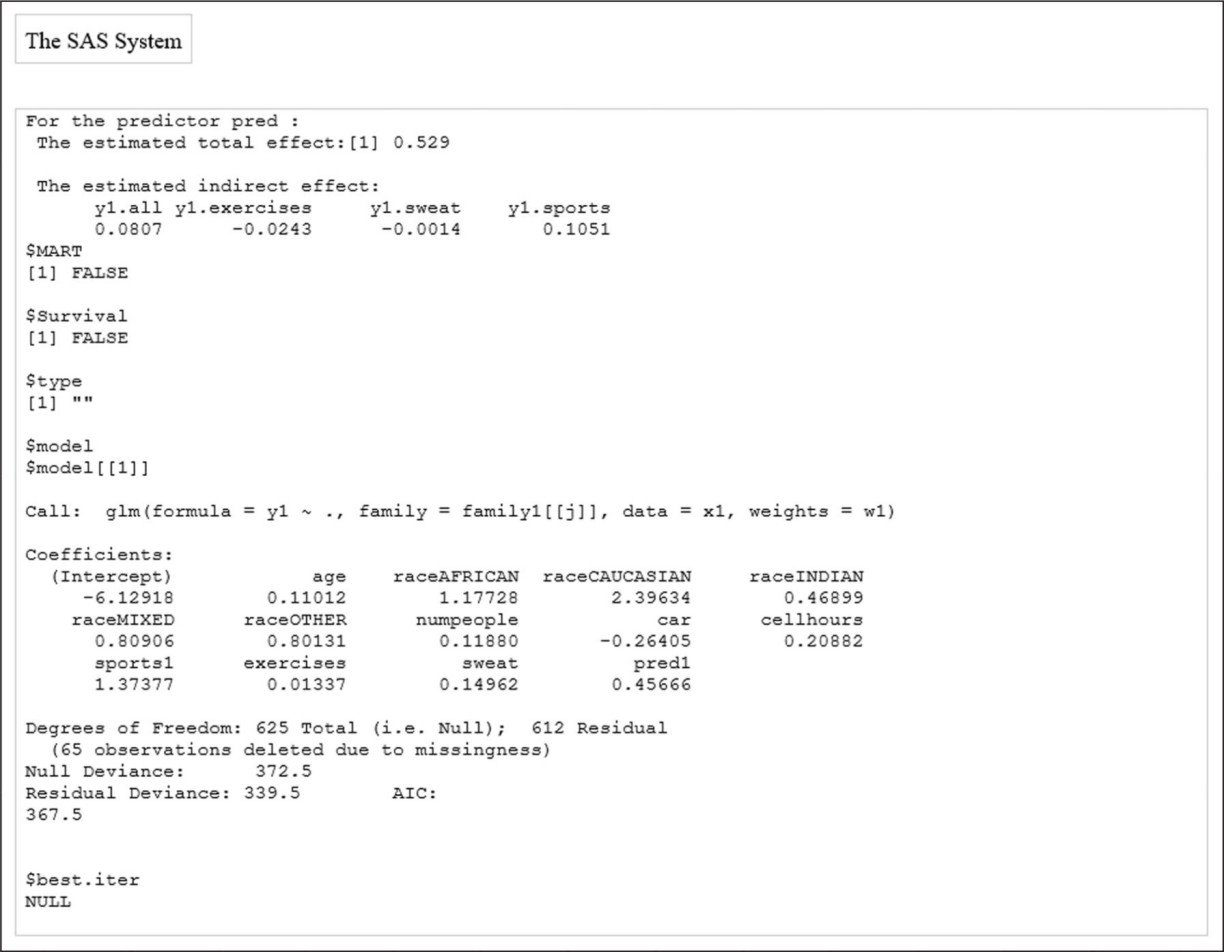
SAS output with the final full model.

The temp.med R data is produced from the Proc_R_med macro and is stored in the path defined by the PATH_R argument. Figure 2 presents the final full model results and the estimated total effect and indirect effects.

#### Proc_R_bootmed Macro

The function bootmed in R uses bootstrap method for mediation effects inferences. The mediation effects, variances, and confidence intervals of the estimated mediation effects are estimated from bootstrap samples.

### Arguments

The Proc_R_bootmed macro is included in the file Proc_R_bootmed.sas. The arguments used in the macro are summarized in Table 3 in addition to Tables 1 and 2. More information on how to define the arguments REFY, MARGIN, D, DISTN, n, n2, NU, NONLINEAR, DF1, TYPE, RE, can be found in [3].

### Implementation

All arguments can be defined in the temp_setup_mma_macro.sas file. The macro programs, Proc_R_bootmed.sas and R_submit_bootmed.sas, are saved in the appropriate location(s) defined in the argument PATH_R.

The macro reads in the R dataset that was created by the Proc_R_dataorg macro, data.bin.RData, and then calls the bootmed function in R to perform the analysis. As a result of the macro, a summary table and a plot with all estimates and confidence intervals are printed in the SAS output window and the plot is save in the file data_bin_plot.png in the location defined in the argument PATH_R. The data_bin_plot.png plot presents the estimated mediation effects with their confidence intervals. The total effect, direct effect, and the indirect effects are displayed. When the argument _RE_ is set as TRUE, the plot and summary are on the estimated relative effects instead.

### Example

#### The dataset

Using the same example as above, the data.bin.RData that was exported from the Proc_R_dataorg macro.

#### Implementation

The code below defines all of the macro arguments and is saved in the Proc_R_bootmed macro. The number of bootstrap iterations is set to be 4 times.


%let rdata=data.bin ;
%let n=2 ;
%let n2=4 ;
%let nonlinear=FALSE ;


Now, execute the %INCLUDE statement to run the SAS program which contains the macro.


%include “&path\Proc_R_bootmed.sas” ;


Lastly, run the Proc_R_bootmed macro.


%Proc_R_bootmed(&pre, &path_r, &rdata, &margin, 
&D, &distn, &refy, &n, &n2, &nu, &nonlinear, 
&df1, &type) ;


#### Results

**Figure 3:**
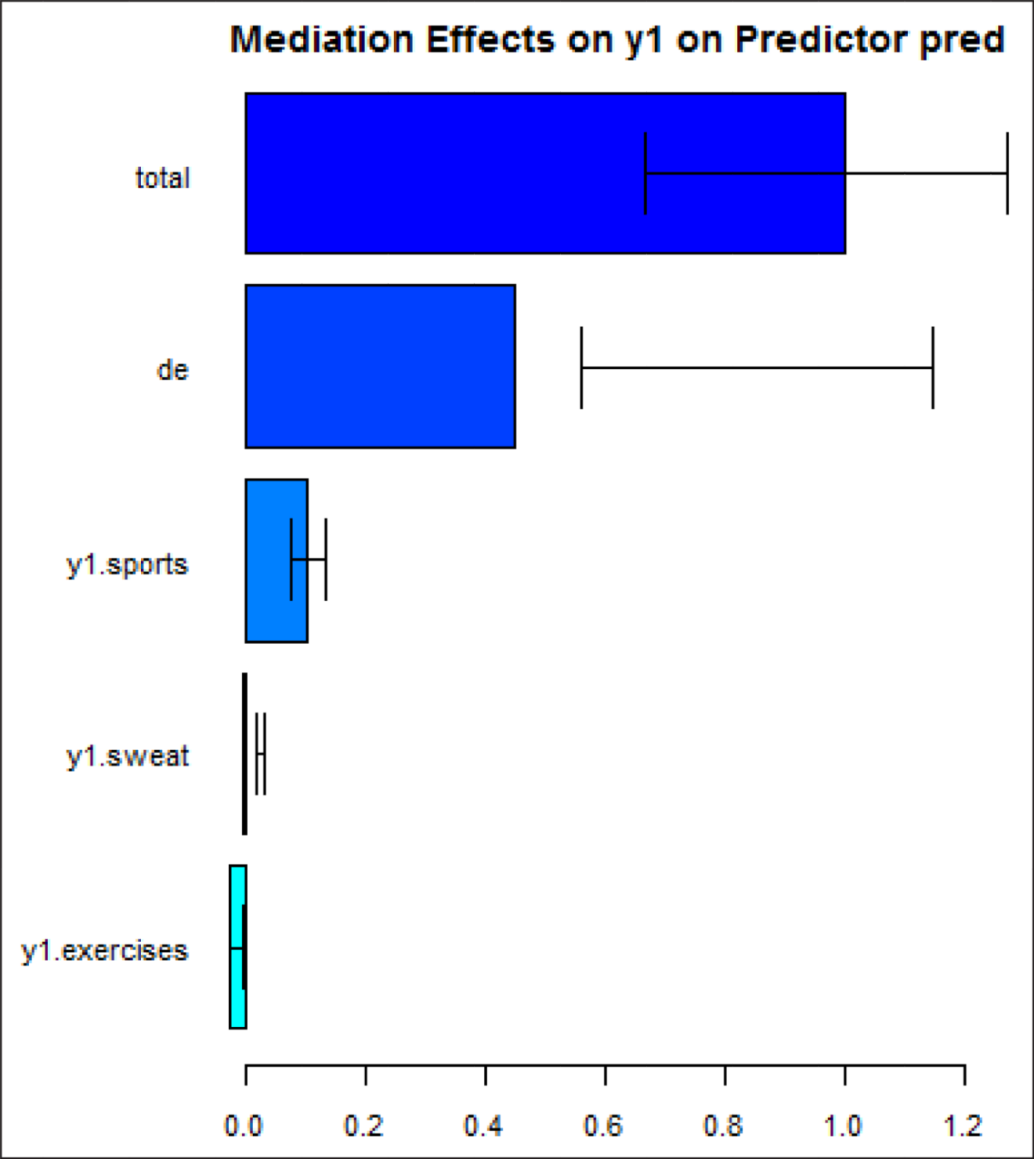
The estimated mediation effects.

**Figure 4:**
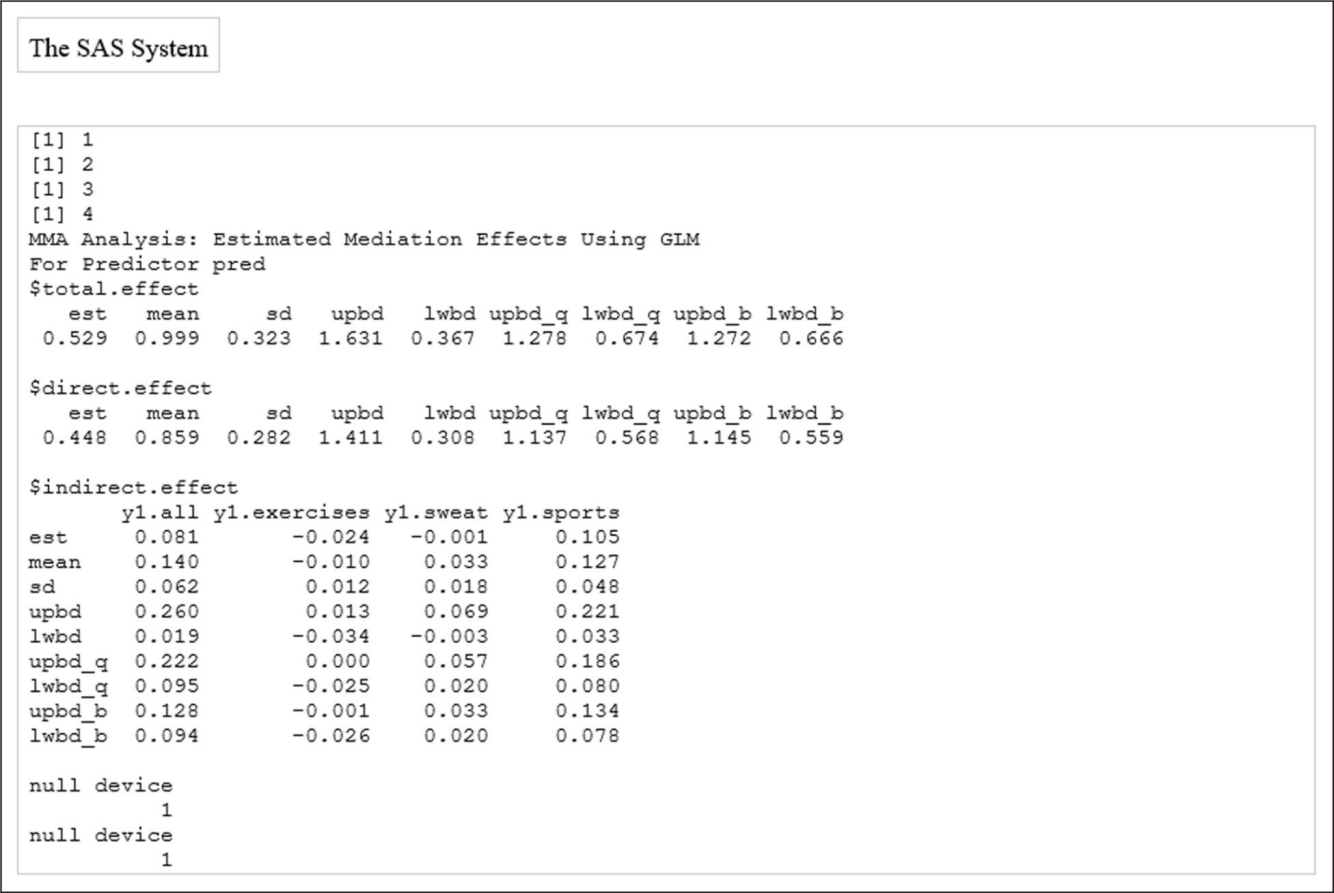
Summary of the bootmed function.

Figure 3 shows the estimated mediation effects for the mediators that were identified: “sports”, “sweat”, and “exercises”. Figure 4 displays the *summary* output. For example, using the quantile confidence interval, “sports” explains about 13% (95% Confidence Interval (3%, 18%)) of the sexual difference in overweight, “sweat” explains about 3.3% (2%, 5.7%) of the sexual difference in overweight, while “exercises” is not significant in explaining the sexual difference in overweight: the confidence interval includes 0.

#### Proc_R_bootmed_plot Macro

The plot function helps illustrate how the predictor relates to the mediator, and how the mediator is related with the response variable. For continuous predictors, the fitted relationship between the predictor and the mediator is plotted. For binary and categorical predictors, the distributions of the mediator at different levels of the predictor are graphed.

### Arguments

The Proc_R_bootmed_plot macro is included in the file Proc_R_bootmed_plot.sas. The arguments used in the macro are summarized in Table 4. More information on how to define the arguments VARI, XLIM, ALPHA, QUANTILE can be found in [3].

### Implementation

As before, all arguments are defined in the temp_setup_mma_macro.sas file. The user must save the macro program, Proc_R_bootmed_Plot.sas and R_submit_bootmed_Plot.sas in the appropriate location(s) defined in the argument PATH_R.

The macro uses the R data that was created by the Proc_R_bootmed macro, data.bin.plot.RData, to summarize the mediation effects. The plot, data_bin_plot2.png, shows the relationship between the outcome and variable which is specified by _VARI_ and between the variable _VARI_, and the predictor (this was defined in the argument PRED of Proc_R_dataorg macro).

### Example

#### The dataset

Using the same example as above, the data.bin.plot.RData was exported from the Proc_R_bootmed macro.

#### Implementation

First, we define the argument that we are interested to examine the relationship between overweight and exercise as well as the relationship between exercise and sex.


%let vari=exercises ;


Then execute the %INCLUDE statement to run the SAS program which contains the macro.


%include “&path\Proc_R_bootmed_Plot.sas” ; 


Lastly, run the Proc_R_bootmed_Plot macro.


%Proc_R_bootmed_plot(&pre, &path_r, &vari, 
&alpha, &quantile, &xlim) ;


#### Results

**Figure 5:**
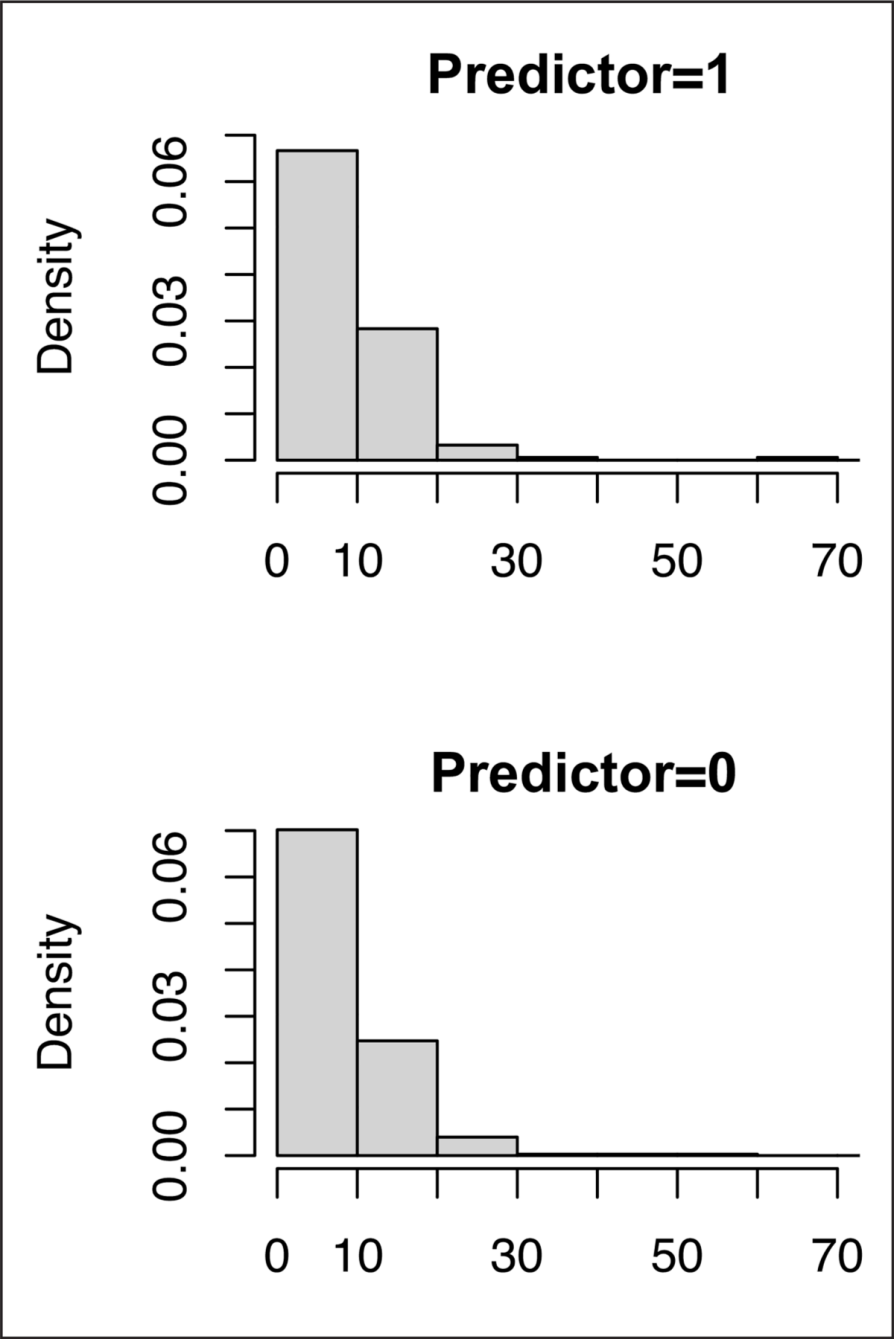
The marginal effects of exercise on overweight and the marginal effect of sex on exercise.

Figure 5 shows the relationship between overweight and exercises and the distributions of exercise (in hours per week) by gender (predictor = 0 for males and = 1 for females). The fitted relationship between overweight and exercise is linear since a generalized linear model was used to model the relationship.

### Quality control

All the functions of the SAS macros were tested to see they produce the desired results by comparing outputs from the SAS results to the mma package in R.

## (2) Availability

### Operating system

The macro can work on Windows operating system.

### Programming language

SAS version 9.22 or above is required. R version 2.14.1 or higher.

### Additional system requirements

An Internet connection is required to install the mma package.

### Dependencies

R packages: gbm, car, gplots, splines, and survival.

### List of contributors

The mma R package was created by Dr. Qingzhao Yu and Dr. Bin Li.

### Software location

***Archive*** (e.g. institutional repository, general repository) (required – please see instructions on journal website for depositing archive copy of software in a suitable repository)

***Name:*** CRAN

***Persistent identifier:*** https://cran.r-project.org/web/packages/mma/

***Licence:*** GPL (>=2)

***Publisher:*** Qingzhao Yu and Bin Li

***Version published:*** 10.3–2

***Date published:*** 05/24/2020

**SAS macros:** Available as supplementary materials to the article.

### Language

SAS, R

## (3) Reuse potential

In this article, we introduce SAS macros to perform multiple mediation analysis utilizing the “mma” R package. These macros allow SAS users to perform multiple mediation analysis within the SAS environment. Thus, for those SAS users that are not familiar with R, this provides them with the tools for mediation analysis. By running these macros, results from the R environment are read into the SAS as SAS database. Users can utilize the bootstrap samples and results, and tools provided by SAS to perform any further analysis.

In addition, the SAS macros provides a template for people to call R packages from the SAS environment. Writers of R package can extend the use of these macros to generate arguments and dataset necessary for running the package in R. Results from R can be similarly read back into SAS for further analysis. As a future research, we will work on creating SAS macros for SAS users that perform multiple mediation analysis for high-dimensional data sets by utilizing the R package, “mmabig” [5] and to perform multilevel mediation analysis using the R package “mlma”.

## Supplementary Material

SAS macros

## Figures and Tables

**Table 1:** Arguments for Proc_R_dataorg Macro.

Arguments	Description	Notes

PATH	Path where the macro is saved.	
PATHD	Path where the SAS dataset is stored.	By default, the SAS dataset will be saved in the “Work” SAS library.
DATA	Name of the SAS dataset that contains the data for mediation analysis.	Needs to read the data into SAS format before implementing the macro.
PATH_R	Path where R dataset and txt file will be stored.	Make sure the path includes only forward slashes (/).
X	The dataset that contains all potential mediators and covariates.	
MEDIATOR	The list of names or the column numbers in x of all potential mediators. If listed, the function tells whether the potential mediator is continuous or categorical by the type and number of unique values of each variable.	The arguments CATMED and BINMED are used only when specific reference groups are needed to be specified (by BINREF and CATREF).
CONTMED	Names or column numbers of continuous mediators in X.	
BINMED	Names or column numbers of binary mediators in X.	
BINREF	Reference group(s) of the potential binary mediators in BINMED.	The default reference group is the first level of the mediator.
CATMED	Names or column numbers of categorical mediators in X.	If the categorical variable has 3 or more groups and numeric values, it must be specified using CATMED to be treated as categorical.
CATREF	Reference groups of the potential categorical mediators in CATMED.	The default is the first level of the mediator.
PREDREF	If the predictor is categorical, the reference group for the predictor.	The default is the first level of the predictor.
JOINTM	Group(s) of variables whose joint effect is of interest.	The first item is the number of groups of joint mediators and the following items identify the column numbers of the mediators in X for each group of joint mediators.
REFY	The reference group for Y if Y is binary.	
ALPHA	The significance level to test if the potential mediators is significant in estimating Y.	The default is ALPHA=0.1.
ALPHA2	The significance level to test if a potential mediator is significantly related with the predictor.	The default is ALPHA2=0.1.
PRED	The vector or matrix of predictor(s).	
Y	The vector or matrix of the outcome variable.	If Y is a survival outcome, then define it using the Surv(time,status) function.
TIME	If Y is a time-to-event outcome, this is the variable in the dataset that indicates follow up time.	
STATUS	If Y is a time-to-event outcome, this is a 0 to 1 indicator identifies no event or event separately.	

**Table 2:** Arguments for Proc_R_med Macro.

Arguments	Description	Notes

MARGIN	The change in predictor when calculating the mediation effects.	The argument is useful only when the predictor is continuous. By default, MARGIN=1.
D	If MART is used, the parameter specifies the “interaction. depth” in gbm function.	The default is D=3.
DISTN	If MART is used for the final full model, the assumed distribution of the outcome.	The default is DISTN=“gaussian” for continuous y and DISTN=“bernoulli” for binary y.
n	The time of resampling in calculating the indirect effects.	The default is n=20.
NU	If MART is used, set the parameter “shrinkage” in gbm function.	The default is nu=0.001.
NONLINEAR	If NONLINEAR=TRUE, MART will be used to fit the final full model in estimating the outcome. Splines with degree freedom DF1 are used to fit the relationship between the predictor and potential mediators.	The default is NONLINEAR=FALSE, a generalized linear model will be used.
DF1	The degrees of freedom in the ns() function when MART is used.	The default is DF1=1.
TYPE	The type of prediction when Y is class Surv.	The default is “risk”.

**Table 3:** Arguments for Proc_R_bootmed Macro.

Arguments	Description	Notes

n2	The number of times of bootstrap resampling.	The default is n2 = 50.
RE	The summary function will also report the summaries of the relative effects, calculated as the “(in)direct effect/total effect” if RE=TRUE.	

**Table 4:** Arguments for Proc_R_bootmed_Plot Macro.

Arguments	Description	Notes

VARI	The name of the variable to plot.	
XLIM	The range of the variable to be plotted.	
ALPHA	For continuous predictor only, to draw the 1-alpha confidence interval of the indirect effect.	
QUANTILE	For continuous predictor only, to draw the alpha confidence interval of the indirect effect based on quantile QUANTILE=TRUE.	
