# Supplementary material for: Using SAS Macros for Multiple Mediation Analysis in R
Source: J Open Res Softw. Author manuscript; Available in PMC 2021 Aug 4. (PMC8336624; doi:10.5334/jors.277)
Supplement: SAS macros [file NIHMS1685015-supplement-SAS_macros.pdf]

```
/* *************************************/
/* File Name: PROC_R_dataorg            */
/* Desciption:                                        */
/* Macro to save data.bin as txt file & */
/* R data                                                   */
/* *************************************/

%macro
proc_R_dataorg(pre,path_r,data,mediator,contmed,binmed,binref,catmed,catref,predref,jointm,refy,alpha,alp
ha2,x,pred,y,time,status) ;
/*write SAS dataset into a working directory*/
data wd ;
set &pre.&data. ;
run;

/*export SAS dataset to txt file*/
proc export data=wd
   outfile="&pathd\&data..txt"
       dbms=TAB replace;
   putnames=YES;
run;

proc iml ;
pre="&pre" ;
path="&path";
path_r="&path_r";
data="&data" ;
mediator="&mediator" ;
contmed="&contmed";
binmed="&binmed";
binref="&binref";
catmed="&catmed";
catref="&catref";
predref="&predref";
jointm="&jointm";
refy="&refy";
alpha="&alpha" ;
alpha2="&alpha2" ;
x="&x" ;
pred="&pred" ;
y="&y" ;
time="&time" ;
status="&status" ;
%INCLUDE  "&path\R_submit_dataorg.sas" ;
%mend proc_R_dataorg ;

/* *************************************/
/* File Name: R_submit.sas              */
/* Desciption:                                        */
/* Run R in SAS                                   */
/* *************************************/
submit pre path_r data mediator contmed binmed binref catmed catref predref jointm refy alpha alpha2 x
pred y time status/R ;

###load pacakges
library(mma)
```

```
library(survival)
data=read.table("&path_r/&data.txt",sep="\t", header = TRUE, strip.white = FALSE)
attach(data)
x=&x
pred=&pred
status=&status
time=&time
y=&y

data.bin<-
data.org(x=x,y=y,pred=pred,mediator=&mediator,contmed=&contmed,binmed=&binmed,binref=&binref,

catmed=&catmed,catref=&catref,predref=&predref,jointm=&jointm,refy=&refy,alpha=&alpha,alpha2=&alph
a2)
save(data.bin, file = "&path_r/data.bin.RData")
out <- capture.output(data.bin)
cat("", out, file="&path_r/data_bin.txt", sep="n", append=TRUE)
detach(data)

summary(data.bin)

endsubmit;

/* ***************************************/
/* File Name: PROC_R_med                 */
/* Desciption:                           */
/* Macro to summarize mediation effects */
/*                                       */
/* R data                                */
/* ***************************************/

%macro Proc_R_med(pre,path_r,rdata,margin,D,distn,refy,n,nu,seed,nonlinear,df,type) ;

proc iml ;
pre="&pre" ;
path_r="&path_r";
rdata="&rdata" ;
margin="&margin" ;
D="&D" ;
distn="&distn" ;
refy="&refy" ;
n="&n";
nu="&nu" ;
seed="&seed";
nonlinear="&nonlinear" ;
df="&df" ;
type="&type" ;
%INCLUDE  "&path_r\R_submit_med.sas" ;

%mend Proc_R_med;

/* ***************************************/
/* File Name: R_submit_med.sas       */
/* Desciption:                           */
/* Run R in SAS - med                */
/* ***************************************/
submit pre path_r rdata margin D distn refy n nu seed nonlinear df type/R ;
```

```
###load pacakges
library(mma)
load("&path_r/&rdata.RData")
temp.med<-
med(data=data.bin,margin=&margin,D=&D,distn=&distn,refy=&refy,n=&n,nu=&nu,seed=&seed,nonlinear=
&nonlinear,df=&df,type="&type")
save(temp.med, file = "&path_r/temp.med.RData")
print(temp.med)
temp.med[["model"]]
endsubmit;

/* ************************************/
/* File Name: PROC_R_bootmed          */
/* Desciption:                        */
/* Macro to summarize mediation effects */
/* and save plot data_bin_plot.png    */
/* R data                             */
/* ************************************/


%macro Proc_R_bootmed(pre,path_r,rdata,margin,D,distn,refy,n,n2,nu,seed,nonlinear,df1,type,RE) ;

proc iml ;
pre="&pre" ;
path_r="&path_r";
rdata="&rdata" ;
margin="&margin" ;
D="&D" ;
distn="&distn" ;
refy="&refy" ;
n="&n";
n2="&n2";
nu="&nu" ;
seed="&seed";
nonlinear="&nonlinear" ;
df1="&df1" ;
type="&type" ;
RE="&RE" ;
%INCLUDE  "&path_r\R_submit_bootmed.sas" ;

%mend Proc_R_bootmed;

/* ************************************/
/* File Name: R_submit_bootmed.sas    */
/* Desciption:                        */
/* Run R in SAS - bootmed             */
/* ************************************/
submit pre path_r rdata margin D distn refy n n2 nu seed nonlinear df1 type RE/R ;
###load pacakges
library(mma)
load("&path_r/&rdata.RData")
data.bin.plot<-
boot.med(data=data.bin,margin=&margin,D=&D,distn=&distn,refy=&refy,n=&n,n2=&n2,nu=&nu,seed=&se
ed,nonlinear=&nonlinear,df1=&df1,type="&type")
png("&path_r/data_bin_plot.png")
summary(data.bin.plot, RE=&RE)
dev.off()
```

```
save(data.bin.plot, file = "&path_r/data.bin.plot.RData")

endsubmit;

/* **************************************/
/* File Name: PROC_R_bootmed_Plot          */
/* Desciption:                               */
/* Macro to save plot data_bin_plot2.png   */
/* R data                                    */
/* **************************************/


%macro Proc_R_bootmed_plot(pre,path_r,vari,alpha,quantile,xlim) ;

proc iml ;
pre="&pre" ;
path_r="&path_r";
vari="&vari" ;
alpha="&alpha" ;
quantile="&quantile" ;
xlim="&xlim" ;
%INCLUDE  "&path_r\R_submit_bootmed_plot.sas" ;

%mend Proc_R_bootmed_plot;

/* **************************************/
/* File Name: R_submit_bootmed.sas    */
/* Desciption:                               */
/* Run R in SAS - bootmed                   */
/* **************************************/
submit pre path_r vari alpha quantile xlim/R ;
###load pacakges
library(mma)
load("&path_r/data.bin.plot.RData")

png("&path_r/data_bin_plot2.png")
plot(data.bin.plot, vari="&vari",xlim=&xlim,alpha=&alpha,quantile=&quantile)
dev.off()

endsubmit;

/* **************************************/
/* File Name: setup_mma_macro          */
/* Desciption:                               */
/* Define Location for current analysis */
/* and define data names all parameters */
/* needed for mma package function      */
/* **************************************/

options formchar="|----|+|---+=|-/\<>*";
proc options option=RLANG value; run; *allows you to run R in SAS;

libname lib "Z:\Documents\LSU\LSU_RESEARCH\Yu\survival" ;
%let path=Z:\Documents\LSU\LSU_RESEARCH\Yu ;
%let pathd=Z:\Documents\LSU\LSU_RESEARCH\Yu\survival; *path where SAS dataset is stored ;
%let data=cgd0 ; *SAS dataset that is saved in the folder specified in pathd;
%let pre=lib.; /*lib here what you named your libname -- you may change it to what your libname is*/
```

```
%let path_r=Z:/Documents/LSU/LSU_RESEARCH/Yu/survival;

/*for data.org */
%let mediator=(1:8) ;
%let contmed=;
%let binmed=;
%let binref= ;
%let catmed= ;
%let catref= ;
%let predref= ;
%let refy= ;
%let alpha=0.4 ;
%let alpha2=0.4 ;
%let x=%str(data[,c(5:12)]) ;
%let pred=%str(data[,4]) ;
%let y=%str(data[,15]) ;
*if y is survival ;
%let time=%str(data$futime) ; *days to last follow-up ;
%let status=%str(ifelse(is.na(data$etime1),0,1)) ; *0 or 1 indicator funciton ;
%let y=%str(Surv(time,status)) ;

%include "&path\Proc_R_dataorg.sas" ;
***data.org macro - save data.bin R data ;
%Proc_R_dataorg(&pre,&path_r,&data,&mediator,&contmed,&binmed,&binref,&catmed,&catref,&predref,&r
efy,&alpha,&alpha2,&x,&pred,&y,&time,&status);

/*for med*/
%let rdata=data.bin ;
%let margin= ;
%let D= ;
%let distn= ;
%let refy= ;
%let n=2 ;
%let nu=;
%let seed=1 ;
%let nonlinear=FALSE;
%let df1= ;
%let type= ;

%include "&path\Proc_R_med.sas" ;

***med macro - print summary and save plot ;
%Proc_R_med(&pre,&path_r,&rdata,&margin,&D,&distn,&refy,&n,&nu,&seed,&nonlinear,&df1,&type)

/*for boot.med */
%let rdata=data.bin ;
%let margin= ;
%let D= ;
%let distn= ;
%let refy= ;
%let n=2 ;
%let n2=4 ;
%let nu= ;
%let seed=1 ;
%let nonlinear=TRUE ;
%let df1= ;
%let type= ;
```

```
%let RE= ;

%include "&path\Proc_R_bootmed.sas" ;

***boot.med macro - print summary and save plot ;
%Proc_R_bootmed(&pre,&path_r,&rdata,&margin,&D,&distn,&refy,&n,&n2,&nu,&seed,&nonlinear,&df1,&typ
e,&RE) ;


/*for boot.med PLOT*/
%let vari=exercises ;
%let alpha= ;
%let quantile= ;
%let xlim= ;

%include "&path\Proc_R_bootmed_plot.sas" ;

***boot.med PLOT macro - print summary and save plot ;
%Proc_R_bootmed_plot(&pre,&path_r,&vari,&alpha,&quantile,&xlim) ;

/* **************************************/
/* File Name: setup_mma_macro            */
/* Desciption:                                         */
/* Define Location for current analysis */
/* and define data names all parameters */
/* needed for mma package function      */
/* **************************************/

options formchar="|----|+|---+=|-/\<>*";
proc options option=RLANG value; run; *allows you to run R in SAS;

libname lib "Z:\Documents\LSU\LSU_RESEARCH\Yu" ;
%let path=Z:\Documents\LSU\LSU_RESEARCH\Yu ;
%let pathd=Z:\Documents\LSU\LSU_RESEARCH\Yu; *path where SAS dataset is stored ;
%let data=weight_behavior ; *SAS dataset that is saved in the folder specified in pathd;
%let pre=lib.; /*lib here what you named your libname -- you may change it to what your libname is*/
%let path_r=Z:/Documents/LSU/LSU_RESEARCH/Yu;

/*for data.org */
%let mediator= ;
%let contmed=c(7:9,11:12) ;
%let binmed=c(6,10) ;
%let binref=c(1,1) ;
%let catmed=5 ;
%let catref=1 ;
%let predref=;
%let jointm= ;
%let refy= ;
%let alpha=0.4 ;
%let alpha2=0.4 ;
%let x=%str(data[,c(2,4:14)]) ;
%let pred=%str(data[,3]) ;
%let y=%str(data[,15]) ;

*if y is survival ;
%let time=%str(data[]) ; *days to last follow-up ;
%let status=%str() ; *0 or 1 indicator funciton ;
```

```
%let y=%str(Surv(time,status)) ;

%include "&path\Proc_R_dataorg.sas" ;
***data.org macro - save data.bin R data ;
%Proc_R_dataorg(&pre,&path_r,&data,&mediator,&contmed,&binmed,&binref,&catmed,&catref,&predref,&j
ointm,&refy,&alpha,&alpha2,&x,&pred,&y,&time,&status);

/*for med*/
%let rdata=data.bin ;
%let margin= ;
%let D= ;
%let distn= ;
%let refy= ;
%let n=2 ;
%let nu=;
%let seed=1 ;
%let nonlinear=FALSE;
%let df1= ;
%let type= ;

%include "&path\Proc_R_med.sas" ;

***med macro - print summary and save plot ;
%Proc_R_med(&pre,&path_r,&rdata,&margin,&D,&distn,&refy,&n,&nu,&seed,&nonlinear,&df1,&type)

/*for boot.med */
%let rdata=data.bin ;
%let margin= ;
%let D= ;
%let distn= ;
%let refy= ;
%let n=2 ;
%let n2=4 ;
%let nu= ;
%let seed=1 ;
%let nonlinear=TRUE ;
%let df1= ;
%let type= ;
%let RE= ;

%include "&path\Proc_R_bootmed.sas" ;

***boot.med macro - print summary and save plot ;
%Proc_R_bootmed(&pre,&path_r,&rdata,&margin,&D,&distn,&refy,&n,&n2,&nu,&seed,&nonlinear,&df1,&typ
e,&RE) ;


/*for boot.med PLOT*/
%let vari=exercises ;
%let alpha= ;
%let quantile= ;
%let xlim=c(1,70) ;

%include "&path\Proc_R_bootmed_plot.sas" ;

***boot.med PLOT macro - print summary and save plot ;
%Proc_R_bootmed_plot(&pre,&path_r,&vari,&alpha,&quantile,&xlim) ;
```

```
/* *************************************/
/* File Name: temp_setup_mma_macro           */
/* Desciption:                                              */
/* Define Location for current analysis */
/* and define data names all parameters */
/* needed for mma package function      */
/* *************************************/

options formchar="|----|+|---+=|-/\<>*";
proc options option=RLANG value; run; *allows you to run R in SAS;

libname lib "   " ; *libname for current analysis put location in “” ;
%let path= ; *path where macro is saved ;
%let pathd=  ; *path where SAS dataset is stored ;
%let data=  ; *SAS dataset that is saved in the folder specified in pathd;
%let pre=lib.; /*lib here what you named your libname -- you may change it to what your libname is or keep
it lib if didn’t change above */
%let path_r=; *path where R dataset and txt file will be stored;
*see https://cran.r-project.org/web/packages/mma/mma.pdf for info on how to define the parameters below
;

***data.org ;

%let mediator= ;
%let contmed= ;
%let binmed= ;
%let binref= ;
%let catmed= ;
%let catref= ;
%let predref= ;
%let jointm= ;
%let refy= ;
%let alpha= ;
%let alpha2= ;
%let x=%str(data[]) ;
%let pred=%str(data[]) ;
%let y=%str(data[]) ;

*if y is survival ;
%let time=%str(data[]) ; *days to last follow-up ;
%let status=%str( ) ; *0 or 1 indicator funciton ;
%let y=%str(Surv(time,status)) ;

%include "&path\Proc_R_dataorg.sas" ;
***data.org macro - save data.bin R data ;
%Proc_R_dataorg(&pre,&path_r,&data,&mediator,&contmed,&binmed,&binref,&catmed,&catref,&predref,&j
ointm,&refy,&alpha,&alpha2,&x,&pred,&y,&time,&status);

***med;

%let rdata=data.bin ;
%let margin= ;
%let D= ;
%let distn= ;
%let refy= ;
```

```
%let n= ;
%let nu=;
%let seed= ;
%let nonlinear=;
%let df= ;
%let type= ;

%include "&path\Proc_R_med.sas" ;

***med macro - print summary and save plot ;
%Proc_R_med(&pre,&path_r,&rdata,&margin,&D,&distn,&refy,&n,&nu,&seed,&nonlinear,&df,&type)

***boot.med ;

%let rdata=data.bin ;
%let margin= ;
%let D= ;
%let distn= ;
%let refy= ;
%let n= ;
%let n2= ;
%let nu= ;
%let seed= ;
%let nonlinear= ;
%let df= ;
%let type= ;
%let RE= ;

%include "&path\Proc_R_bootmed.sas" ;

***boot.med macro - print summary and save plot ;
%Proc_R_bootmed(&pre,&path_r,&rdata,&margin,&D,&distn,&refy,&n,&n2,&nu,&seed,&nonlinear,&df,&type
,&RE) ;

/*for boot.med PLOT*/
%let vari= ;
%let alpha= ;
%let quantile= ;
%let xlim= ;

%include "&path\Proc_R_bootmed_plot.sas" ;

***boot.med PLOT macro - print summary and save plot ;
%Proc_R_bootmed_plot(&pre,&path_r,&vari,&alpha,&quantile,&xlim) ;
```
